# Atherosclerosis: an overview of mouse models and a detailed methodology to quantify lesions in the aortic root

**DOI:** 10.1530/VB-23-0017

**Published:** 2024-04-04

**Authors:** Jamie I van der Vaart, Robin van Eenige, Patrick C N Rensen, Sander Kooijman

**Affiliations:** 1 Division of Endocrinology, Department of Medicine, Leiden University Medical Center, Leiden, The Netherlands; 2 Einthoven Laboratory for Vascular and Regenerative Medicine, Leiden University Medical Center, Leiden, The Netherlands

**Keywords:** atherosclerosis, hyperlipidemia, mouse models, quantification, aortic root

## Abstract

Cardiovascular disease, the primary cause of human mortality globally, is predominantly caused by a progressive disorder known as atherosclerosis. Atherosclerosis refers to the process of accumulation of cholesterol-enriched lipoproteins and the concomitant initiation of inflammatory processes in the arterial wall, including the recruitment of immune cells. This leads to the formation of atherosclerotic plaques, initially causing a thickening of the arterial wall and narrowing of arteries. However, as plaque formation progresses, atherosclerotic plaques may become unstable and rupture, leading to a blood clot that blocks the affected artery or travels through the blood to block blood flow elsewhere. In the early 1990s, emerging gene editing methods enabled the development of apolipoprotein E knockout (*Apoe^−/−^*) and low-density lipoprotein receptor knockout (*Ldlr^−/−^*) mice. These mice have been instrumental in unraveling the complex pathogenesis of atherosclerosis. Around the same time, human APOE*3-Leiden transgenic mice were generated, which were more recently cross-bred with human cholesteryl ester transfer protein (CETP) transgenic mice to generate APOE*3-Leiden.CETP mice. This model appears to closely mimic human lipoprotein metabolism and responds to classic lipid-lowering interventions due to an intact ApoE–LDLR pathway of lipoprotein remnant clearance. In this review, we describe the role of lipid metabolism and inflammation in atherosclerosis development and highlight the characteristics of the frequently used animal models to study atherosclerosis, with a focus on mouse models, discussing their advantages and limitations. Moreover, we present a detailed methodology to quantify atherosclerotic lesion area within the aortic root region of the murine heart, as well as details required for scoring atherosclerotic lesion severity based on guidelines of the American Heart Association adapted for mice.

## Introduction

Cardiovascular diseases (CVDs) are currently the leading cause of death globally. Alarmingly, the total number of CVD cases has nearly doubled from 271 million in 1990 to 523 million 2019 (1). The main underlying cause of CVDs is atherosclerosis, a progressive disorder characterized by a thickening of arterial walls and narrowing of the arteries due to the formation of atherosclerotic plaques, that is, accumulation of cholesterol, macrophages, and cell debris in the intima of the arteries (2, 3). The main risk factors for the development of atherosclerotic CVD (asCVD) are combined hyperlipidemia (i.e. high levels of plasma triglycerides and cholesterol) and inflammation (4).

In this review, we will first describe the pathogenesis of atherosclerosis, followed by an overview of different animal models to study atherosclerosis, with a focus on mouse models. We will conclude this review with a detailed description of an established method in our laboratory to characterize atherosclerosis within the aortic root area of the mouse heart.

## Atherosclerosis development

Traditionally, atherosclerosis has been regarded as a lipid-driven disease, where the retention of lipoproteins in the intima of arteries was considered to be the main causal factor. However, later observations showed that circulating monocytes infiltrate the developing plaques, revealing an inflammatory component of the disease (5). In the following sections, we will describe the role of both aspects in the development of atherosclerotic plaques.

### Lipoprotein metabolism

Lipoproteins are essential carriers of lipids, transporting them in the lymphatics and circulation between tissues and organs. They typically consist of a hydrophobic core of nonpolar lipids (i.e. triglycerides and cholesteryl esters), surrounded by a hydrophilic shell containing phospholipids, unesterified cholesterol, and apolipoproteins (6). At least four classes of lipoproteins can be distinguished based on their size, lipid composition, and apolipoprotein composition, namely chylomicrons, very-low-density lipoprotein (VLDL), low-density lipoprotein (LDL), and high-density lipoprotein (HDL) (6).

**Figure 1:**
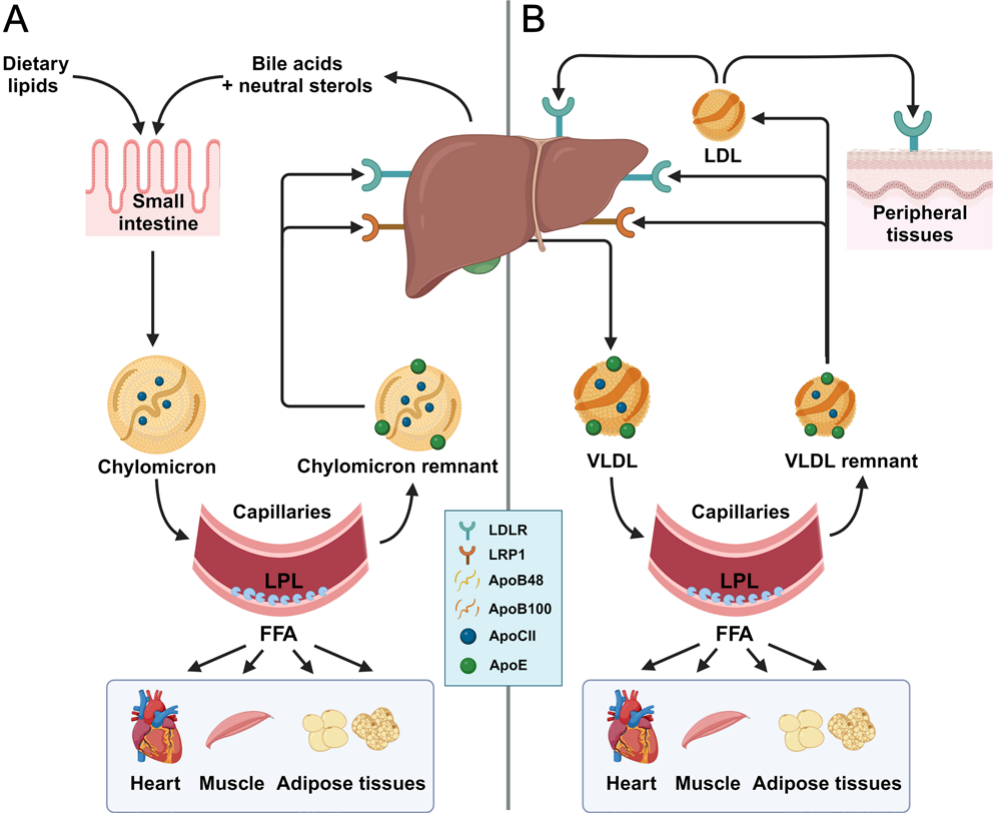
A schematic overview of ApoB-containing lipoprotein metabolism. See text for details. Apo, apolipoprotein; FFA, free fatty acid; LDL, low-density lipoprotein; LDLR, LDL receptor; LPL, lipoprotein lipase; LRP1, LDL receptor-related protein 1; VLDL, very-low-density lipoprotein.

Chylomicrons are formed in the small intestine (Fig. 1A) (4, 6). Starting in the gastrointestinal tract, dietary triglycerides are broken down by gastric lipase and pancreatic lipase into 2-monoacylglycerols and fatty acids, which are absorbed by enterocytes. Here, they are readily reconverted into triglycerides with the aid of monoacylglycerol acyltransferase (MGAT) and diacylglycerol transferase (DGAT) (7). Simultaneously, dietary cholesterol is taken up by enterocytes and largely esterified into cholesteryl esters by acyl-CoA cholesterol acyltransferase (ACAT). Within the endoplasmic reticulum of enterocytes, microsomal triglyceride transfer protein (MTP) subsequently combines lipid droplets formed from triglycerides, cholesteryl esters, and phospholipids with apolipoprotein (Apo) B48 (ApoB48; i.e. 48% of the ApoB transcript) to form pre-nascent chylomicron particles (8, 9). Ultimately, chylomicrons are secreted into the lymph and enter the circulation via the thoracic duct, where they acquire exchangeable apolipoproteins primarily produced by hepatocytes. This includes ApoC2, which is an essential cofactor for lipoprotein lipase (LPL). When LPL is activated on the endothelial surface of capillaries, it catalyzes the hydrolysis of triglycerides within chylomicrons, allowing for the uptake of the liberated fatty acids by the underlying tissue as an energy source (10). During delipidation, the generated chylomicron remnants acquire increasing amounts of ApoE, which allows the remnants to be cleared from the circulation primarily by hepatocytes via binding of ApoE to the LDL-receptor (LDLR) and the LDLR-related protein 1 (LRP1), followed by endocytosis.

The liver can use the lipids taken up from chylomicron remnants for oxidation, or use them together with *de novo* synthesized lipids and cholesterol to generate VLDL (Fig. 1B). Here, MTP facilitates the fusion of lipid droplets with ApoB100 (i.e. 100% of the ApoB transcript), and other apolipoproteins, including ApoA5, ApoC1, ApoC2, ApoC3, and ApoE, also associate with the newly produced VLDL (6, 11). Once in the circulation, LPL hydrolyzes the triglycerides within VLDL (similar to triglycerides within chylomicrons), after which the delipidated VLDL remnants are also cleared primarily via binding of ApoE to the LDLR and LRP1 on hepatocytes within the liver. Part of the VLDL remnants escape clearance by the liver and are further lipolyzed, leading to the formation of essentially triglyceride-free LDL (4). As LDL also no longer contains ApoE, it can only be cleared via interaction of ApoB100 with the LDLR on hepatocytes or on other organs that require cholesterol, such as the adrenals. As the affinity of ApoB100 for the LDLR is much lower than that of ApoE, and there is no interaction with LRP1, LDL has a much longer retention time in the circulation than VLDL and thus tends to accumulate. The metabolism of chylomicrons, VLDL, and LDL is summarized in Fig. 1.

Both the liver and the intestine synthesize and secrete lipid-poor ApoA1, the precursor of HDL (6). Once in the circulation, ApoA1 acquires excess phospholipids that are liberated from chylomicrons and VLDL during LPL-mediated lipolysis, mediated via the phospholipid transfer protein (PLTP) (12). This nascent pre-HDL also takes up cholesterol from peripheral organs via diffusion and via the ATP-binding cassette transporter A1 (ABCA1) and G1 (ABCG1), forming cholesterol-enriched pre-HDL. Subsequently, lecithin cholesterol acyltransferase (LCAT) catalyzes the esterification of free cholesterol into cholesteryl esters, producing mature HDL, which can interact with the scavenger receptor class B type 1 (SR-B1) on hepatocytes. This results in the selective uptake of cholesteryl esters from HDL by the liver (6, 12). Cholesteryl esters can also be transferred from HDL to ApoB-containing lipoproteins in exchange for triglycerides with the aid of cholesteryl ester transfer protein (CETP) (13, 14). Eventually, these cholesteryl esters partly end up in the liver via interaction of ApoE with the LDLR and LRP1, or ApoB100 with the LDLR. Regardless of the route, cholesteryl esters taken up by hepatocytes are hydrolyzed within lysosomes into cholesterol. Here, cholesterol can be temporarily stored in hepatocytes as cholesteryl esters after re-esterification or it can be secreted into the bile, either directly by transport via ABCG5 and ABCG8, or indirectly after conversion into bile acids, which is the major route of biliary cholesterol secretion (15, 16). Bile acids released into the gut facilitate the emulsification and absorption of dietary lipids, after which the majority (~95%) of the bile acids is reabsorbed (‘enterohepatic circulation’), with the remainder excreted via feces (17).

### Dyslipidemia and inflammation: two risk factors in atherosclerosis development

**Figure 2:**
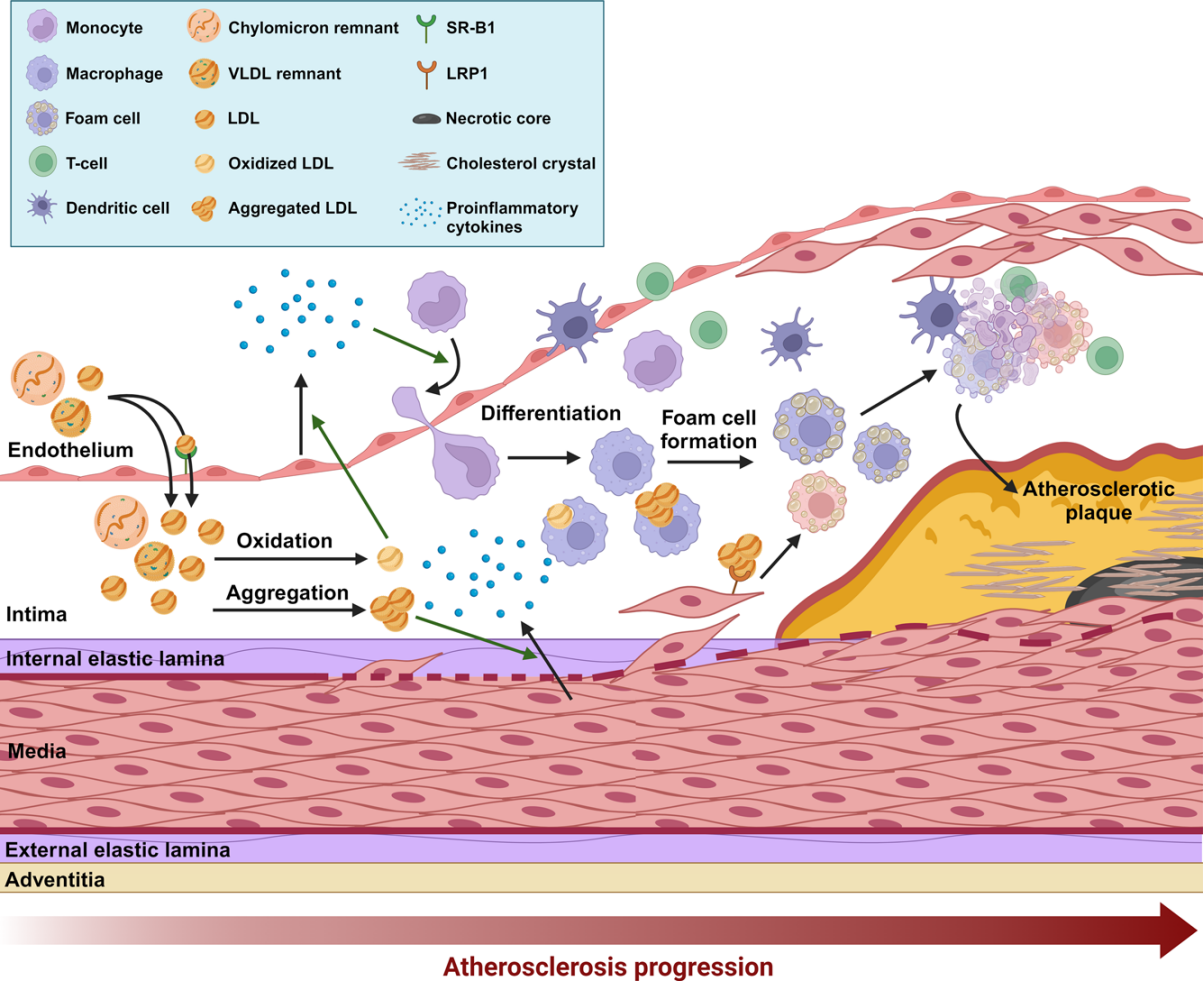
Schematic overview of atherosclerotic plaque development. See text for explanation. Green arrows indicate stimulation of the indicated process. LDL, low-density lipoprotein; LRP1, LDL receptor-related protein 1; SR-B1, scavenger receptor class B type 1; VLDL, very-low-density lipoprotein.

Under certain conditions, such as metabolic syndrome, VLDL can be overproduced by the liver and/or the processing and removal of ApoB-containing lipoproteins (i.e. chylomicrons, VLDL, and LDL) can be impaired. This results in the accumulation of triglycerides and cholesteryl esters carried within these lipoproteins in the circulation, also known as combined hyperlipidemia, which poses a major risk for the development of coronary artery disease (CAD). ApoB-containing lipoproteins are pro-atherogenic because they can cross the endothelium of artery walls via paracellular transport (‘paracytosis’), thereby entering the arterial intima (Fig. 2) (4). Per particle, VLDL is considered to be more pro-atherogenic than LDL (18). Nonetheless, LDL likely contributes most to atherosclerosis development as it outnumbers VLDL in humans, and it can cross the endothelium not only via paracytosis but also via SR-B1-mediated transcytosis (19). Once in the intima, LDL can be oxidized by enzymatic or nonenzymatic reactions (20). In addition, LDL can aggregate within the intima, thereby increasing their affinity for arterial proteoglycans, promoting their retention (21). Interestingly, the susceptibility of LDL to aggregate has been shown to associate with future cardiovascular deaths (22).

Besides hyperlipidemia, inflammatory pathways play an integral part in the initiation and progression of atherosclerosis. Although the triggers needed to start the inflammatory cascade in atherogenesis has/have not been fully elucidated, it is generally believed that one of the first steps involves the activation of endothelial cells and smooth muscle cells lining the arterial vessel wall. This leads to an increase in the expression of adhesion molecules, including intercellular adhesion molecule 1 (ICAM-1) and vascular cell adhesion molecule 1 (VCAM-1). Activated endothelial cells also start overexpressing chemoattractant proteins, such as monocyte chemoattractant protein 1 (MCP1), chemokine (C–C motif) ligand 5 (CCL5), interferon gamma-induced protein 10 (IP-10), and chemokine (C–X3–C motif) ligand 1 (CX3CL1) (23). As a result, immune cells, including monocytes, T-cells, and dendritic cells, are recruited into the intima (3, 23). The secretion of proinflammatory cytokines (e.g. macrophage-colony stimulating factor; MCSF and interleukin 1 beta; IL-1β) by endothelial cells additionally stimulates the newly recruited monocytes to differentiate into macrophages (24). In reaction to these various inflammatory cytokines (e.g. MCSF, MCP1, and IL-1β) the liver produces C-reactive protein (CRP), which in turn stimulates inflammatory reactions in the vascular endothelium, thereby further promoting plaque formation (25). In line with this, circulating levels of CRP have been shown to be predictive for the long-term outcome of patients with CAD and the risk of complications as a consequence of atherosclerosis (26, 27).

Once differentiated, macrophages in the intima upregulate the expression of scavenger receptors, such as scavenger receptor A (SRA) and cluster of differentiation 36 (CD36), to engulf mainly modified (i.e. oxidized or aggregated) LDL and lipoprotein remnants (3). In addition, smooth muscle cells can break through the internal elastic lamina where they can take up primarily aggregated LDL via LRP1 (28). The accumulated cholesterol in both macrophages and smooth muscle cells is stored as cholesteryl esters in cytoplasmic lipid droplets or in unesterified form in lysosomes and cell membranes. As the cholesterol levels in macrophages and smooth muscle cells continue to rise, the cells are converted into so-called foam cells.

Foam cells form the first basis of an atherosclerotic lesion, called a ‘fatty streak’, and accelerate lesion development by secreting proinflammatory cytokines and stimulating the retention of lipoproteins (3). Further accumulation of unesterified cholesterol in the foam cells ultimately leads to cell death and the formation of cholesterol crystals (29, 30, 31). In an early atherosclerotic lesion, apoptotic cells are cleared by macrophages and other phagocytic cells through a process called efferocytosis (32). However, as the plaque formation progresses, efferocytosis becomes impaired, leading to the accumulation of foam cells, apoptotic cells, and cell debris, resulting in the formation of a necrotic core within the atherosclerotic plaque (32). In parallel, an overlying matrix of collagen, proteoglycans, and smooth muscle cells, referred to as a ‘fibrous cap’, is formed, which is crucial to maintain lesion integrity (33). However, foam cells also release matrix metalloproteinases (MMPs) and other enzymes that degrade the extracellular matrix, thereby weakening the fibrous cap and increasing plaque vulnerability, specifically increasing the risk of rupture (34, 35). Upon rupture, the highly thrombotic material from the plaque’s interior is exposed to the circulation, thereby initiating a coagulation cascade (33), which may cause an acute thrombotic occlusion of a coronary or carotid artery, and may lead to a myocardial infarction or a stroke, respectively. In fact, plaque rupture is the most frequent finding in autopsy studies of patients with sudden cardiac death or myocardial infarction (36, 37).

### Animal models to study atherosclerosis

What should be clear by now is that the pathophysiology of atherosclerosis is complex, multifactorial, and dependent on numerous immuno-metabolic interactions that cannot easily be studied in cell culture models. Furthermore, given the chronic, progressive character of the disease and the limited availability of visualization techniques, studying pathogenesis in humans, let alone studying the anti-atherogenic potential of novel strategies, is very challenging. For that reason, as early as 1908, Alexander Ignatowski established the first animal model to study atherosclerotic plaque formation using rabbits fed a diet enriched in fat and cholesterol through supplementation with egg yolk and milk (38). Since then, rabbits became a popular model within the atherosclerosis field and were essential for the discovery and description of the underlying pathology of atherosclerosis. For example, Nikolai Anichkov was the first to report in 1913 that hypercholesterolemia causes atheromatous changes in the vascular wall of rabbits (39). In addition, he was the first to observe multiple cell types present within atherosclerotic lesions, only later to be identified as macrophages, lymphocytes, and smooth muscle cells (40). Even though the initial suggestion that circulating lipids exist in complexes with proteins was made by Michel Machebouef in 1929 (41), it was not until the 1950s, when John W Gofman and Frank Lindgren were able to isolate different lipoprotein fractions using an ultracentrifuge protocol and showed that these lipoproteins build up in the vessel wall to form atherosclerotic lesions (42, 43).

In the early 1990s, emerging gene editing techniques enabled the generation of genetically modified mice as models to study atherosclerosis, and mice have ever since been the favored experimental animal to study atherosclerosis. The choice of mice as a model for atherosclerosis may be somewhat surprising, as no known inbred mouse strain exists that spontaneously develops atherosclerosis. This can be explained by the fact that mice have very low (VL)DL-cholesterol levels, and the majority of circulating cholesterol (approximately 80%) is found within HDL, while in humans most of the circulating cholesterol (approximately 75%) is within (V)LDL (44). Nevertheless, the use of mice in research has several advantages. For example, mice are small in size and thus have low housing costs, they rapidly reproduce, and if susceptible, they need a relatively short period of time to develop atherosclerosis (45, 46). With respect to the latter, Paigen *et al.* investigated ten different mouse inbred strains to show that C57BL/6 mice are most susceptible to developing diet-induced atherosclerosis (47). Given that the genetic map of inbred mouse strains was relatively well defined, this discovery even allowed for the identification of certain genetic links to atherosclerosis susceptibility in the years to follow (48, 49, 50). In mice, atherosclerotic plaques typically do not rupture. However, a stability index may be determined as a measure of plaque vulnerability (see section ‘Determining atherosclerotic lesion composition’). Also, the developmental stages of atherosclerotic plaques can be scored, ranging from early fatty streaks to clinically dangerous plaques, which is elaborated on in the section ‘Scoring atherosclerosis severity’.

#### ApoE knockout model

Among the first mouse models for atherosclerosis obtained through genetic engineering was the ApoE knockout model (*Apoe^−/−^*). *Apoe^−/−^* mice were generated through homologous recombination in embryonic stem cells, as reported in 1992 (51). As described in the section ‘Lipoprotein metabolism’, ApoE is essential for the uptake of triglyceride-rich lipoprotein remnants from the circulation via the LDLR and LRP1 present on hepatocytes (52, 53). Since hepatic uptake of remnants is thus abrogated in *Apoe^−/−^* mice, these mice exhibit hyperlipidemia with circulating total cholesterol levels up to 10–15 mM on a regular chow diet, and exceeding 25 mM on a cholesterol-containing Western-type diet (51, 54). In comparison, wildtype C57BL/6 mice do not exhibit cholesterol levels above 2.5 mM, of which the majority represents HDL cholesterol. As a result, *Apoe^−/−^* mice develop advanced atherosclerosis already at 2–3 months of age even on a regular chow diet (55).

In *Apoe^−/−^* mice, atherosclerotic lesions mainly develop in the valve sinus, including the coronary arteries, but also in the aortic root, aortic branches, the carotid artery, mesenteric artery, renal and pulmonary arteries (56, 57). Interestingly, strain-dependent susceptibility to atherosclerosis, as discussed before, translates into differences in the topography of lesion development. For example, *Apoe^−/−^* mice on a C57BL/6 background are more prone to develop atherosclerosis in the aortic root region, whereas a 129S6 background yields plaques mostly in the aortic arch, and on a DBA/2J background plaques develop in both locations (58). In addition, there seems to be a sex-dependent susceptibility to atherosclerosis with larger and more advanced lesions in young, but not old, female *Apoe^−/−^* mice compared with age-matched male mice on a normocholesterolemic diet (59). This is different from what is observed in humans, where males typically develop atherosclerotic lesions at a younger age than females in line with the atheroprotective effects of estrogen that have also been reported in *Apoe^−/−^* mice (60, 61).

Since their development, *Apoe^−/−^* mice have been widely used to discover new therapeutic drugs and drug targets for atherosclerosis, especially in the field of anti-inflammatory strategies. For example, ablation of IL-1β in *Apoe^−/−^* mice was found to reduce atherosclerosis development by approximately 30% (62). This finding appeared translationally relevant as the more recent Canakinumab Anti-inflammatory Thrombosis Outcome Study (CANTOS) trial showed that canakinumab, a monoclonal antibody targeting IL-1β, lowers recurrent cardiovascular events in patients with a history of myocardial infarction (63). It should be noted, though, that immunomodulatory interventions do increase the risk for infections, as also became apparent with canakinumab (63); therefore, such strategies may only be suitable for specific subpopulations of patients, for example, those at a very high risk of recurrent cardiovascular events. ApoE also has functions beyond lipoprotein metabolism. For example, *Apoe* is expressed by hematopoietic cells, where it regulates proliferation rate (64). ApoE also suppresses the type I inflammatory responses, as concluded from elevated pro-inflammatory cytokine expression in lipopolysaccharide (LPS)-stimulated *Apoe*^−/−^ mice but not in other hypercholesteremic models (65). Furthermore, ApoE in macrophages controls the cholesterol efflux to HDL, which contributes to the impaired reverse cholesterol transport as observed in *Apoe*^−/−^ mice (66, 67).

#### LDLR knockout model

The genetic link between the LDLR and familial hypercholesterolemia was suggested already in 1974 when Brown and Goldstein showed that cells derived from patients with familial hypercholesterolemia lack high-affinity receptors for binding LDL (68). Therefore, by targeting the LDLR with homologous recombination of embryonic stem cells, an *Ldlr^−/−^* mouse model was created and reported in 1993 (69, 70). *Ldlr^−/−^* mice are hypercholesteremic on a regular chow diet, albeit to a lesser extent than observed in *Apoe^−/−^* mice, with total cholesterol levels of 5–8 mM (70). However, when fed a cholesterol-enriched diet, cholesterol levels can increase up to 40 mM, resulting in accelerated atherosclerosis development compared to the same mice on a regular chow diet (71). Nevertheless, the topography of plaques in the *Ldlr^−/−^* mouse on a C57BL/6 background is highly comparable to that in *Apoe^−/−^* mice on the same background (71, 72). As for *Apoe^−/−^* mice, there is surprisingly little information about sexual dimorphism in atherosclerosis development in *Ldlr^−/−^* mice and the published studies are typically not powered to address this issue. Nevertheless, a relatively large study suggested larger atherosclerotic lesions in female compared to male *Ldlr^−/−^* mice on a C57BL/6J background, but not on an FVB/NJ background (73, 74). Interestingly, a recent study suggested that the use of older *Ldlr^−/−^*mice more closely resembles the human situation with more advanced atherosclerotic lesions and the presence of age-associated T and B cells both in atherosclerotic plaques and in the circulation (75).

Similarly to *Apoe^−/−^* mice, the *Ldlr^−/−^* mice are widely employed to study atherosclerosis and have some advantages over the *Apoe^−/−^* mouse model. First, in contrast to *Apoe^−/−^* mice where cholesterol mainly accumulates within chylomicrons and VLDL, the *Ldlr^−/−^* mice accumulate cholesterol mostly as LDL particles, generating a more human-like lipid profile (51, 76). Secondly, the atherosclerotic phenotype of *Ldlr^−/−^* mice is in large caused by elevated lipid levels and is less affected by effects beyond those on lipoprotein metabolism, as is the case for *Apoe^−/−^* mice (77).

As many lipid-lowering drugs directly or indirectly rely on LDLR-mediated lipoprotein uptake, the effect of those drugs cannot be studied in *Ldlr^−/−^* mice, and this applies to some extent also for *Apoe^−/−^* mice as they lack the main ligand of the LDLR. These drugs include statins, which are used by more than 200 million people around the world to lower CVD risk. The reason for this is that, although inhibition of cholesterol biosynthesis via 3-hydroxy-3-methyl-glutaryl-coenzyme A (HMG-CoA) reductase is the primary target of statins, a large part of the cholesterol-lowering effect of statins is mediated via a compensatory hepatic upregulation of the LDLR (78, 79). Another example of a drug class that is not effective in the *Ldlr^−/−^* mouse is the recently developed proprotein convertase subtilisin/kexin type 9 (PCSK9) inhibitors (78). PCSK9 is a protein that binds to the LDLR and facilitates its lysosomal degradation, thereby ultimately attenuating the hepatic clearance of lipoproteins remnants from the circulation (80). Inhibition of PCSK9 thus lowers LDL levels preventing the degradation of the LDLR. Altogether, this indicates that the *Ldlr^−/−^* mouse model is less suitable to test the efficacy, toxicity, or mechanism of action of newly developed lipid-lowering therapeutics (45).

#### APOE*3-Leiden.CETP model

Interestingly, even before the description of *Apoe^−/−^* and the *Ldlr^−/−^* mice, yet another mouse model for atherosclerosis was reported based on the discovery of the APOE*3-Leiden mutation in a Dutch family with a genetic form of dysbetalipoproteinemia (81). This APOE*3-Leiden mutation, characterized by a tandem repeat of seven amino acids just outside the LDLR binding domain (82), caused a decrease in the affinity of APOE*3 for the LDLR, resulting in a slower clearance of chylomicron and VLDL remnants from the circulation (71). A genomic construct containing *APOE, APOC1,* and all regulatory elements was isolated from a proband of this family and used to generate mice transgenic for human APOE*3-Leiden and *APOC1* as described in 1991 (83).

A major advantage of the APOE*3-Leiden mice is that they still express endogenous ApoE, and thus have a functional ApoE–LDLR clearance pathway. Only when fed a cholesterol-containing Western-type diet, APOE*3-Leiden mice become hyperlipidemic and display total cholesterol levels of approximately 10–20 mM divided over (V)LDL and HDL (83). In 2006, the first report of a crossbreeding between APOE*3-Leiden mice and human CETP transgenic mice appeared, resulting in APOE*3-Leiden.CETP double-transgenic mice (84). While not expressed in wild-type mice, CETP mediates the transfer of cholesteryl esters from HDL to (V)LDL in exchange for triglycerides in humans, the main effect being a reduction in HDL-cholesterol and increasing levels of (V)LDL-cholesterol. Importantly, these mice appear to respond similar to humans when it comes to the lipid-lowering and atheroprotective effects of, for example, statins (85), fibrates (86), niacin (87), and the PCSK9 antibody alirocumab (88, 89) mainly due to the presence of the APOE*3-Leiden transgene, but also to the HDL-raising effects of the CETP inhibitors torcetrapib (86) and anacetrapib (90). Moreover, we have employed APOE*3-Leiden.CETP mice to show that newly developed experimental drugs for obesity and diabetes, such as a fibroblast growth factor 21 (FGF21) analogue (91), a glucagon-like peptide 1 (GLP-1) receptor agonist (92), and the combination of a GLP-1 receptor agonist with a glucose-dependent insulinotropic polypeptide (GIP) receptor agonist, also have lipid-lowering and atheroprotective effects (93).

Similar to the APOE*3-Leiden mice,APOE*3-Leiden.CETP mice are hyperlipidemic only when fed a cholesterol-containing Western-type diet, with total cholesterol levels reaching 10–30 mM depending on the amount of cholesterol in the diet (typically ranging between 0.10% and 0.25%). It should be noted, however, that in 10–15% of Western-type diet-fed APOE*3-Leiden and APOE*3-Leiden.CETP mice, total cholesterol levels remain below the threshold of approximately 7 mM to develop atherosclerosis after a typical run-in period of 4 weeks (often referred to as ‘low-responders’ or ‘non-responders’), and are therefore not included for randomization in experiments (94, 95). Also, cholesterol exposure (i.e. the area under the curve from circulating total cholesterol levels plotted against the time on a Western-type diet) is quite an accurate predictor for the average atherosclerotic lesion size in the aortic root region of APOE*3-Leiden and APOE*3-Leiden.CETP mice. We typically aim for a cholesterol exposure of 250–300 mM*weeks, corresponding with an average total atherosclerotic lesion size of approximately 1.0–1.5 × 10^5^ µm^2^ per cross section of the aortic valve area in APOE*3-Leiden.CETP mice. In that situation, lesions of all severity types (as detailed in the section ‘Scoring atherosclerosis severity’) can be identified, allowing for studying both anti- and pro-atherogenic potential of (pharmacological) interventions (89, 91). In practice, this would require a treatment period of approximately 14 weeks, assuming a run-in period of 4 weeks and an average total cholesterol level of 15 mM. Concerning the topography of lesions, the different anatomical sites at which APOE*3-Leiden.CETP mice develop atherosclerosis have, to our knowledge, not been formally mapped. However, they have been mapped for the APOE*3-Leiden mice, which develops lesions in the aorta, proximal coronary arteries, aortic root, aortic arch, and large vessels upon a high-fat diet (96). We expect a comparable topography for APOE*3-Leiden.CETP mice.

A limitation of the APOE*3-Leiden.CETP mouse model is that only female mice develop atherosclerosis upon Western-type diet feeding. Although the exact reason remains unclear, male APOE*3-Leiden mice fail to increase hepatic VLDL production in response to a Western-type diet and typically have a more rapid VLDL clearance rate as compared to their female counterparts (97). As a consequence, in male mice, lipids tend to accumulate in the liver rather than in the circulation. This phenotypic feature, combined with the fact that male C57BL/6J mice are generally more prone to develop diet-induced obesity when compared to female mice (98, 99), actually makes male APOE*3-Leiden.CETP mice on their C57BL/6J background a very suitable model for studying lipid-modulating interventions in metabolic dysfunction-associated steatotic liver disease (MASLD), formerly known as nonalcoholic fatty liver disease (NAFLD). For example, we have shown that diet-induced MASLD in male APOE*3-Leiden.CETP mice can be prevented by long-acting FGF21 (100) and combined GLP1R/GIPR agonism (101).

With the identification of triglycerides as an independent risk factor for coronary heart disease (102), novel medication is being developed aimed at accelerating triglyceride removal from the circulation. To study the anti-atherogenic effects of triglyceride-lowering strategies, the presence of the APOE–LDLR clearance pathway appeared critical for the correct translation of findings to humans. For example, a recent study by Sui *et al*. showed that the clinically used β3-receptor agonist mirabegron markedly accelerates atherosclerotic plaque formation in *Apoe^−/−^* and *Ldlr^−/−^* mice as related to accelerated LPL-mediated triglyceride-derived fatty acid uptake by thermogenic adipose tissue, based on which the authors raised concerns about its use in patients (103). However, this effect is likely an artifact of the mouse models used as increased delipidation of triglyceride-rich lipoproteins leads to an accelerated formation of lipoprotein remnants, which are only pro-atherogenic when not cleared from the circulation. As anticipated, we therefore more recently found that mirabegron does not accelerate atherosclerosis development in APOE*3-Leiden.CETP mice, due to effective removal of VLDL remnants via the intact ApoE–LDLR pathway (104). Nonetheless, since thermogenic adipose tissue in humans primarily expresses the β2-adrenergic receptor rather than the β3-adrenergic receptor as in mice (105), mirabegron does not activate human thermogenic adipose tissue at the therapeutic dose of 50 mg (105), and 12 weeks of daily dosing therefore does not affect circulating cholesterol levels in humans (106).

The APOE*3-Leiden.CETP model thus appears to be a relevant model for evaluating the effects of modulation of lipid metabolism on atherosclerosis development. It is particularly relevant for the evaluation of compounds within the pipeline of pharmaceutical companies that are expected to directly or indirectly stimulate LPL activity.

#### Emerging models in atherosclerosis research

In the previous sections we have highlighted the most frequently used mouse models in atherosclerosis research, while there are many other available mouse models, for instance mice expressing both human *APOB* and *CETP* (107). Emerging genetic techniques furthermore allow for specific targeting of atherosclerosis-related genes without the need of germline alterations (108, 109). For instance, adeno-associated viruses (AAVs) have been used to overexpress PCSK9 in the liver and thereby stimulate hepatic LDLR degradation in mice (108), while others have exploited antisense oligonucleotides (ASOs) to directly reduce hepatic *Ldlr* expression (110). Other promising techniques, such as a vaccine against PCSK9, directly target atherosclerosis-related proteins by inducing an immune response. In APOE*3-Leiden.CETP mice, vaccination against PCSK9, resulted in a reduction of plasma lipids levels, lowered systemic and vascular inflammation, and less atherosclerotic lesions in the aorta (111).

While mice have been an essential tool in understanding the molecular mechanisms behind atherosclerotic plaque formation and progression, as well as in identifying novel treatment modalities, gene manipulation is required to induce atherosclerosis, and the absence of rupture events and sexual dimorphisms may limit their translational value. Other animal models are also employed to study atherosclerosis, such as zebrafish. Compared to mice, zebrafish have an even smaller body size, develop more rapidly, and produce a large number of offspring, allowing for affordable large-scale drug-screening experiments. This is why zebrafish have recently become a popular species in research, also in the field of atherosclerosis research, as reviewed by Tang *et al*. (112) However, while these models seem to work well to study the role of specific proteins in lipoprotein metabolism and can be used for drug screening, so far, atherosclerosis development seems to be limited to early lesions (113).

Lastly, recent advances have been made to model heart diseases *in vitro*, including the use of human organoids (i.e. heart-on-a-chip), and 3D-cell printing techniques (114, 115). These *in vitro* techniques are ideal for drug testing and determining therapies in personalized medicine approaches while simultaneously avoiding the use of animals in research. Moreover, they have already been employed to study more complex disease modalities such as arrhythmia (116), cardiac fibrosis (117), myocardial infarction, and ischemia and reperfusion injury (114, 118). However, they have not yet been used to study atherosclerosis development, as it remains difficult to model the interaction between metabolic organs, such as the adipose tissue and the liver, on atherosclerosis development, especially given that such metabolic organs are under neuronal and hormonal control.

## Atherosclerosis quantification in the aortic root area of mouse hearts

Naturally, choosing the right mouse model is the first step in designing an experiment to study atherosclerosis. Equally important, however, is the type of quantification and analysis of atherosclerosis development, which can offer technical challenges. In the following sections, we will describe in detail key steps and considerations when collecting, processing, and analyzing atherosclerotic lesions within the aortic root area of mice. This includes a detailed method for the quantification of lesion size and composition as established in our laboratory. In addition, we provide a method for scoring atherosclerotic lesion severity based on guidelines of the American Heart Association adapted for mice.

### Sample preparation

The first step in the quantification of atherosclerotic lesions is collecting and preparing samples. Before heart collection, hearts may be perfused with ice-cold phosphate buffered saline to remove any remaining blood cells from the heart. It is not advisable to use cervical dislocation for euthanasia as this would disturb the aortic root morphology. This is even more relevant when the aorta itself is also collected, for example, for *en face* atherosclerosis analysis within the aorta itself.

**Figure 3:**
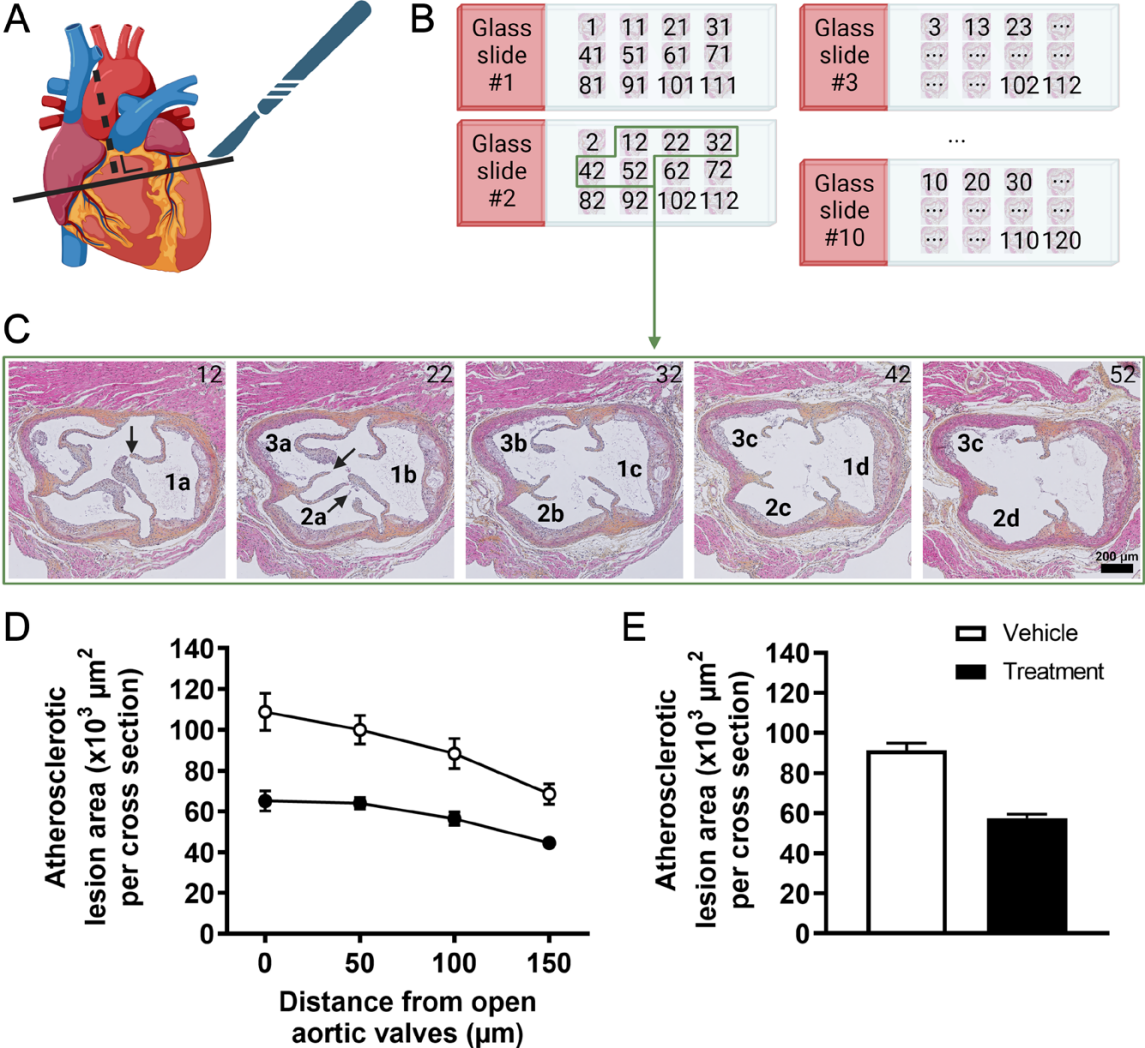
Schematic overview of the process of quantifying atherosclerotic lesion size within the aortic root area of the heart. (A) Collected hearts are cut along an axis perpendicular to the aorta, and are subsequently paraffin-embedded. (B) 120 serial cross sections (5 µm thickness) through the aortic root area are divided over 10 glass slides as depicted. (C) In this example, glass slide #2 from B was stained using hematoxylin–phloxine–saffron, and images of cross sections 12, 22, 32, 42, and 52 are shown. The three aortic valve regions are (arbitrarily) numbered; arrows depict the opening of aortic valve leaflets. In this example as explained in the main text, we would quantify atherosclerotic lesion size in cross sections 12, 22, 32, and 42 for aortic valve region #1, labeled 1a–d, and in cross section 22, 32, 42, and 52 for regions #2 and #3, labeled 2a–d and 3a–d, respectively. Combined with data of the other mice, (D) the absolute atherosclerotic lesion area can subsequently be plotted as a function of the distance from open aortic valves, from which (E) the average atherosclerotic lesion area can be derived. Data depicted in D and E are semi-randomly generated and serve as an example only.

The collected hearts can be processed using standard methods for paraffin embedding, that is, the samples are fixed in a 4% paraformaldehyde for a duration of 24–48 h and subsequently dehydrated in 70% ethanol. The hearts can then be cut using a regular razor blade along the axis perpendicular to the aorta (Fig. 3A), after which the heart tissue containing the aortic root area (i.e. the top one-third) is embedded in paraffin, with the cutting surface positioned on the base of the mold. This ensures that all three (occasionally only two due to a genetic irregularity) aortic valve leaflets will be visible within the same histological cross sections.

In a recent statement, the American Heart Association recommended serial cross-sectioning of the aortic root area from the origin of the aortic valves to the ascending aorta (119). Although no further standard criteria are available, we typically prepare cross sections at a thickness of 5 µm throughout the aortic valve region. Next, starting with a cross section located roughly 50 µm before the appearance of the first open aortic valve leaflet, we recommend dividing 120 serial cross sections over ten separate glass slides in an arrangement where each adjacent cross section on a glass slide differs by 50 µm (Fig. 3B). This arrangement ensures that corresponding cross sections on adjacent glass slides differ by only 5 µm, allowing for a direct comparison of separate glass slides (e.g. to compare different stainings).

### Quantification of atherosclerotic lesion size

After deparaffination, atherosclerotic lesions can be visualized on one glass slide of each mouse using a general staining, such as hematoxylin–phloxine–saffron. To keep atherosclerotic lesion quantification consistent, many research groups use the aortic valve leaflets as a landmark. To this end, the cross section at which that valve leaflet opens is identified. We do this for each aortic valve leaflet separately. Although the number of sections that are analyzed varies between research groups, we typically quantify the lesion sizes within the corresponding valve region in that cross section, and in the subsequent three cross sections, meaning that we are covering a total distance of 150 µm (Fig. 3C). The quantification itself can be done by manually drawing a region of interest around each lesion within each aortic valve region using open-source software such as ImageJ (National Institutes of Health), and determining the absolute lesion area in µm². Please note that in this process of manually delineating regions of interest, researchers should be blinded where possible, and images should be analyzed in a random order to minimize bias.

With the acquired data, one is able to express the absolute atherosclerotic lesion area as a function of the distance of the open aortic valves (i.e. the total lesion area of all aortic root areas combined at 0 µm, 50 µm, 100 µm, and 150 µm distance of open aortic valves; Fig. 3D). From this, the average atherosclerotic lesion area can be calculated by averaging the lesion area at the four distances (Fig. 3E).

### Determining atherosclerotic lesion composition

Given the complexity and heterogeneity of atherosclerotic plaque, insight into compositional changes between groups provides additional insights to solely the lesion size. Atherosclerotic lesion composition (e.g. smooth muscle cell, collagen, and macrophage area %) can be determined using similar methods as described for the hematoxylin–phloxine–saffron staining. As mentioned before, ideally, glass slides adjacent to the general staining are used for such specific stainings as this allows for the most direct comparison with the general staining. After staining and digitalizing a glass slide, one can similarly manually draw regions of interest around the atherosclerotic lesions, again in four cross sections for each aortic valve region separately, starting at the opening of the corresponding aortic valve leaflet. An alternative approach may be to align images of each cross section to images of the corresponding cross section of the general staining, for example using OpenCV for Python (120). This would allow for the re-usage of the regions of interest that were delineated for the general staining with only minimal changes needed, which would save valuable time required for manually delineating entirely new regions of interest. We have recently published a Python script that uses OpenCV for such alignment that can be used for this purpose (121). Whichever method was used to acquire regions of interest around the atherosclerotic lesions for the specific staining, one can next determine the positively stained area within the atherosclerotic lesions, for example by manual or preferably automated color thresholding using software such as ImageJ. Subsequently, the positively stained area % can be obtained by dividing the total positively stained area by the total lesion area. These data may additionally be used to calculate a stability index as a measure for morphological plaque vulnerability. Such an index can be obtained per plaque by dividing the sum of smooth muscle cell area and collagen area by the macrophage area (91, 122).

Naturally, atherosclerotic lesion composition is highly dependent on natural compositional discrepancies between mild and severe lesions. When lesion size differs between treatment groups, it can therefore be insightful to first score atherosclerotic lesion severity, as described in the next section, and to stratify the lesion composition based on the severity. We recommend comparing the atherosclerotic lesion composition and stability index between groups within the compositionally heterogeneous type III lesions only.

### Scoring atherosclerosis severity

In mice, atherosclerotic lesions can be categorized as mild lesions (type I–III) or severe lesions (type IV–V) based on the guidelines of the American Heart Association adapted for mice as originally described by Wong *et al.* (123). This provides insight into whether a treatment improves overall lesion severity, as related to, or independent from changes in atherosclerotic lesion size or composition. Here, the same hematoxylin–phloxine–saffron-stained lesions can be scored as were used for the quantification of atherosclerotic lesion size.

**Figure 4:**
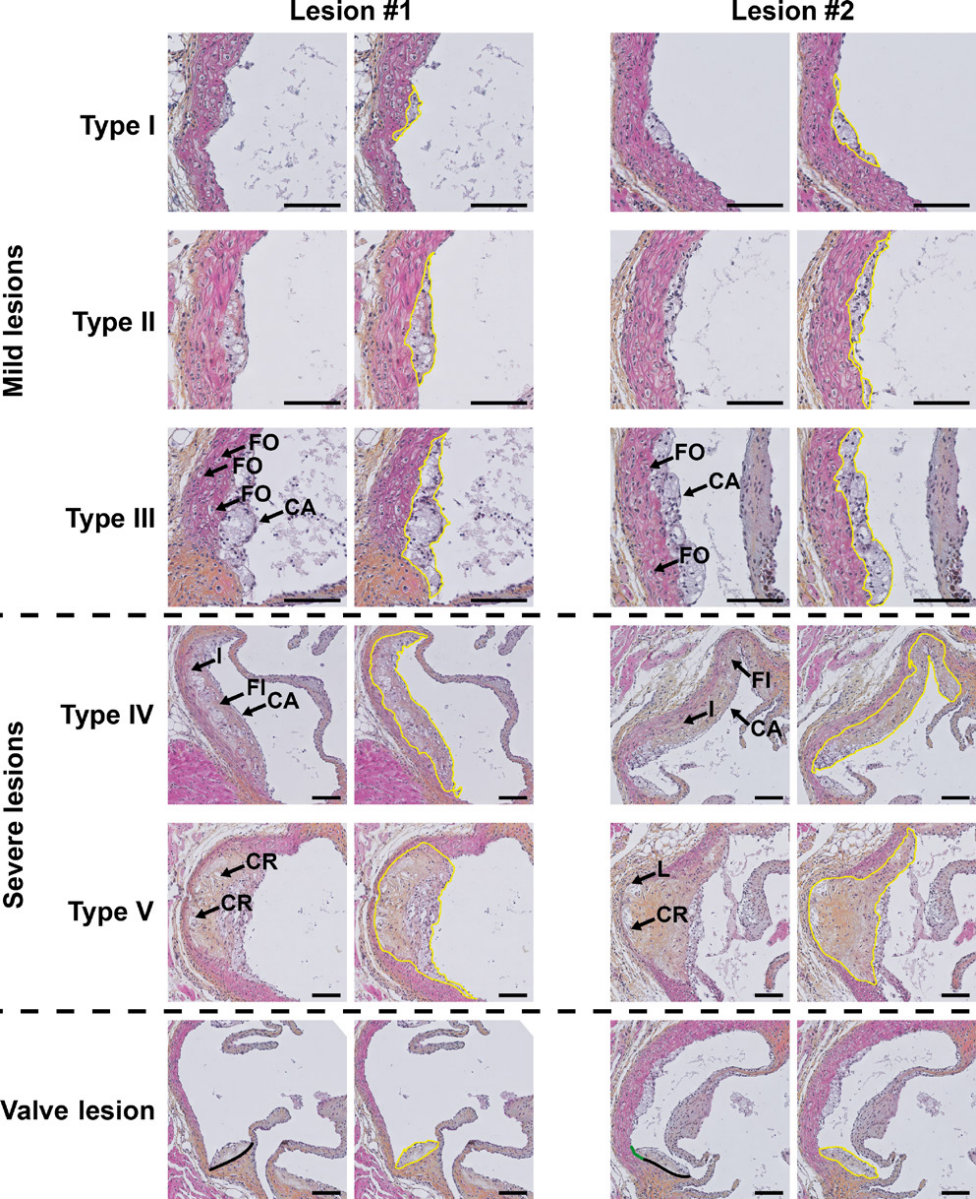
Representative images of aortic valve regions stained with hematoxylin–phloxine–saffron, depicting lesions classified in different severities. Atherosclerotic lesions are categorized into mild lesions (type I–III) and severe lesions (type IV–V). In addition, lesions can be categorized as ‘valve lesions’. Of each lesion type, two lesions are shown in duplicate; yellow lines indicate the circumference of each lesion. Type I, early fatty streak: a maximum of ten foam cells are visible within the intima per cross section. Type II, regular fatty streak: more than ten foam cells are visible within the intima per cross section. Type III, mild plaque: foam cells extend into the media (indicated with ‘FO’) and/or the lesion is covered by a fibrotic cap (indicated with ‘CA’). Type IV, moderate plaque: a progressively advancing lesion that infiltrates into the media (indicated with ‘I’), and is accompanied by fibrosis (indicated with ‘FI’) without architectural loss. Type V, severe plaque: the media is severely damaged. Elastic lamina are broken (indicated with ‘L’), and cholesterol crystals (indicated with ‘CR’), mineralization and/or necrosis can be present. Valve lesion, categorized as such when >50% of the base is located on the root of an aortic valve; black lines indicate (the part of) the base of the valve that is located on the aortic valve root, green lines indicate the remainder of the base of the valve, if any. Scale bars indicate 200 µm.

Mild lesions are early fatty streak-like lesions, characterized by the presence of foam cells within the intima. Here, type I and II lesions are defined as having a maximum or more than ten foam cells per cross section, respectively. Lesions are categorized as type III when foam cells are also localized within the media, and/or the lesion is covered by a fibrotic cap. In contrast, severe lesions are more progressive lesions. Specifically, type IV lesions have infiltrated into the media and are accompanied by fibrosis, but the overall architecture remains intact. However, in type V lesions, the media is severely damaged. In addition, elastic lamina are broken, and cholesterol crystals, mineralization, and/or necrosis can be present. Figure 4 depicts representative images of lesions categorized according to these criteria.

When scoring lesions according to abovementioned criteria, an additional class of lesions, so-called valve lesions, can be identified. Valve lesions are defined as having >50% of their base positioned on the root of a valve, as Fig. 4 exemplifies. It is relevant to distinguish valve lesions from other lesions when scoring atherosclerosis severity, as the morphology and development of these valve lesions usually differ from the other lesions. For that reason, we recommend treating valve lesions as a distinct group instead of scoring them as type I–V. However, it is important to note that we do not treat valve lesions differently in the quantification of atherosclerotic lesion size. Lastly, for each aortic valve region, the number of diseased-free cross sections (i.e. cross sections without any visible lesion or valve lesions) can be counted and reported.

## Concluding remarks

Taken together, asCVD is caused by an interplay between lipids and inflammation. Given the significant burden that atherosclerosis forms on society, multiple animal models have been developed to study atherosclerosis development and to find novel therapeutic strategies to prevent the pathology. In this review, we have discussed the advantages and disadvantages of three most popular models. What should be clear is that it is of critical importance to choose the correct mouse model for correct interpretation of experiments. For example, the presence of the APOE–LDLR clearance pathway in mice is crucial to study the effects of cholesterol- and triglyceride-lowering medication on atherosclerosis development. Using *Apoe^-/-^* or *Ldlr^-/-^* mice to evaluate lipid-lowering strategies can therefore yield results that are opposite of what can be expected in humans. However, both models are very suitable to study inflammation in atherosclerosis development, while the APOE*3-Leiden.CETP model seems most suited to study the lipid-modulating effects of interventions on atherosclerosis development. Given the rapid development of novel genetic techniques and the emergence of zebrafish as experimental units, we anticipate large steps can be made in the field of atherosclerosis research in the coming years. While a suitable animal model to study atherosclerosis is essential, the quantification and analysis method is of equal importance. In this review, we have therefore provided a comprehensive description of all steps required to quantify atherosclerosis development within the aortic root region of the heart of mice, including a detailed description for scoring atherosclerosis severity based on the guidelines of the American Heart Association adapted for mice. This description may help standardize the way results of atherosclerosis experiments are presented in the literature.

## Declaration of interest

The authors declare that there is no conflict of interest that could be perceived as prejudicing the impartiality of the study reported.

## Funding

This work was supported by the Royal Netherlands Academy of Sciences (CVON-GENIUS-II) to PCNR. SK is supported by the Dutch Heart Foundationhttp://dx.doi.org/10.13039/100002129 (2017T016).

## Authors contribution statement

JIVDV and RVE: writing – original draft; RVE, SK and PCNR: writing – review and editing, supervision, funding acquisition.
